# Twitchy, the *Drosophila* orthologue of the ciliary gating protein FBF1/dyf-19, is required for coordinated locomotion and male fertility

**DOI:** 10.1242/bio.058531

**Published:** 2021-08-06

**Authors:** Suzanne H. Hodge, Amy Watts, Richard Marley, Richard A. Baines, Ernst Hafen, Lindsay K. MacDougall

**Affiliations:** 1 School of Biological Sciences, Faculty of Biology, Medicine and Health, University of Manchester, Manchester M13 9PT, UK; 2 Division of Neuroscience and Experimental Psychology, School of Biological Sciences, Faculty of Biology, Medicine and Health, University of Manchester, Manchester Academic Health Science Centre, Manchester M13 9PL, UK; 3 Institute of Molecular Systems Biology, Swiss Federal Institute of Technology (ETH) Zürich, 8093, Zürich, Switzerland

**Keywords:** Cilia, Distal appendage, Transition fibre proteins, Spermiogenesis, *Drosophila*

## Abstract

Primary cilia are compartmentalised from the rest of the cell by a ciliary gate comprising transition fibres and a transition zone. The ciliary gate allows the selective import and export of molecules such as transmembrane receptors and transport proteins. These are required for the assembly of the cilium, its function as a sensory and signalling centre and to maintain its distinctive composition. Certain motile cilia can also form within the cytosol as exemplified by human and *Drosophila* sperm. The role of transition fibre proteins has not been well described in the cytoplasmic cilia.

*Drosophila* have both compartmentalised primary cilia, in sensory neurons, and sperm flagella that form within the cytosol. Here, we describe phenotypes for twitchy the *Drosophila* orthologue of a transition fibre protein, mammalian FBF1/*C. elegans* dyf-19. Loss-of-function mutants in *twitchy* are adult lethal and display a severely uncoordinated phenotype. *Twitchy* flies are too uncoordinated to mate but RNAi-mediated loss of twitchy specifically within the male germline results in coordinated but infertile adults. Examination of sperm from twitchy RNAi-knockdown flies shows that the flagellar axoneme forms, elongates and is post-translationally modified by polyglycylation but the production of motile sperm is impaired. These results indicate that twitchy is required for the function of both sensory cilia that are compartmentalised from the rest of the cell and sperm flagella that are formed within the cytosol of the cell. Twitchy is therefore likely to function as part of a molecular gate in sensory neurons but may have a distinct function in sperm cells.

## Abstract (precis)

**Summary:** Primary cilia are compartmentalised from the rest of the cell by a molecular gate. We show that the *Drosophila* orthologue of a molecular gate protein functions in both compartmentalised sensory cilia and ‘cytosolic’ sperm flagella.

## INTRODUCTION

Primary cilia are hair-like microtubule based projections that extend from the cell (reviewed in Ishikawa and Marshall, 2011). Primary cilia concentrate receptors and ion channels allowing them to respond to changes in the cellular environment and to act as sensory and signalling centres. However, cilia can also be motile and they probably originated from the motile cilia, known as flagella, present in the last eukaryotic common ancestor (Mitchell, 2017). This function is retained in the flagella of extant unicellular organisms such as *Chlamydomonas,* and in the male gametes of many multicellular organisms, such as mammals and the fruit fly *Drosophila*.

Cilia consist of a microtubule core, the axoneme, which grows from the basal body (Jana et al., 2018). Ciliogenesis begins when the centriole, with its symmetrical arrangement of microtubules, matures into a basal body and attaches to the plasma membrane. Initial extension of microtubule doublets from the basal body produces a specialised region, the transition zone. In primary cilia this forms part of a ‘ciliary gate’ that compartmentalises the cilium from the rest of the cell and regulates the movement of large molecules (reviewed in Garcia-Gonzalo and Reiter, 2017; Reiter et al., 2012). Since primary cilia lack other organelles such as ribosomes, large proteins and complexes required for the assembly and function of the cilium must be actively imported from the cell body (Avidor-Reiss and LeRoux, 2015). These complexes include the intraflagellar transport (IFT) proteins that are required to move cargo such as tubulin subunits and hence extend the cilium beyond the cell surface. IFT complexes assemble outside the cilium and must be actively imported through the ciliary gate. Structurally the ciliary gate has been associated with two structures: the transition zone and transition fibres. The Y-links or ‘champagne-glass’ structures of the transition zone (described above) anchor the microtubule axoneme to the ciliary membrane and act as a diffusion barrier (Williams et al., 2011). The second region, consisting of the transition fibres lies below the transition zone and links the basal body to the plasma membrane, demarcating the cilium from the rest of the cell. These arise from the distal appendages and form as the centriole matures into a basal body. Proteomic (Tanos et al., 2013) and super resolution microscopy studies (Bowler et al., 2019; Yang et al., 2018) in mammalian cells have identified several molecular components of the distal appendages/transition fibres and the structures they form at the base of the cilia. In these structures FBF1 localises to a matrix (Yang et al., 2018) or outer ring (Bowler et al., 2019) facilitating its interactions with molecules in the vicinity of the basal body. This location is compatible with a role in gating the movement of cargo such as IFT proteins and transmembrane receptors between the cilium and the rest of the cell (Yang et al., 2018). Y-links and transition fibres have been difficult to visualise in the cilia of *C. elegans* (Blacque and Sanders, 2014; Nechipurenko and Sengupta, 2017) and *Drosophila* (Jana et al., 2018; Ma and Jarman, 2011; Riparbelli et al., 2012; reviewed in Lapart et al., 2020) and only some of the molecular components appear to be evolutionary conserved (Wei et al., 2015). Of these, however, the *C. elegans* FBF1 orthologue dyf-19, localises to the ciliary base and facilitates the ciliary import of assembled IFT particles (Wei et al., 2013).

Most cells in mammals are decorated with one or more cilia (usually less than 10 µm in length) and defective ciliary genes may disrupt the function of multiple organs (reviewed in Garcia-Gonzalo and Reiter, 2017). Longer, single motile cilia are also found in male sperm cells where defective ciliary genes may cause male infertility. In *Drosophila*, in contrast, cilia are restricted to just two cell types, the type I sensory neurons and the motile flagellum of the sperm (Gogendeau and Basto, 2010; Jarman, 2002). These two types of cilia differ in their function and composition but also in how they are formed. In sensory neurons the cilium extends from a basal body and is compartmentalised from the rest of the cell. In contrast, the sperm basal body is attached to the nucleus and the flagellum matures within the cytosol.

The *Drosophila* type I sensory neurons provide sensory and proprioceptive feedback via specialised external sense organs and internal chordotonal organs (Gogendeau and Basto, 2010). The external sense organs are present on the surface of the fly where they are particularly visible as the individual bristles that are deflected by external movements and the hair plates and dome-shaped campaniform sensilla that, together with chordotonal organs, detect body movements. As a result of this restricted distribution, flies with mutations in ciliary components, such as IFT complexes, are viable but uncoordinated.

In *Drosophila* sperm cells, the motile flagellum initially arises as a small primary cilium that projects from the cell (reviewed in Avidor-Reiss and Leroux, 2015; Fabian and Brill, 2012). The basal body elongates to form a ‘giant centriole’ that invaginates, pulling the primary cilium into the cell where it attaches to the nucleus and is enclosed by the plasma membrane to form a ciliary cap. The giant centriole then participates in the subsequent meiotic divisions that form the haploid spermatocyte. Following meiosis, the sperm develop as cysts of 64 interconnected sperm cells encased in somatic head and tail cyst cells, in a process known as spermiogenesis. This results in the formation of an extended and motile cilium (usually referred to as a flagellum) of nearly 2 mm in length that fills most of the length of the coiled testes. The mature sperm bundles are stripped of any unneeded organelles and cytoplasm and each spermatid becomes encased in its own plasma membrane in a process called individualization. These individualised sperm bundles are then coiled in the basal region of the testes where the head cyst cell embeds into the terminal epithelium. The individual, motile sperm are released into the lumen of the testes and swim into the seminal vesicles for storage prior to release on mating.

While the polymerisation of tubulin to assemble the axoneme occurs within the ciliary cap, which maintains a constant length of ∼2 µm, maturation of the axoneme takes place in the cytoplasm. As the axoneme extends, the transition zone, found adjacent to the basal body in primary cilia, migrates distally to separate the elongating distal tip in the ciliary cap from the rest of the axoneme exposed to the cytosol (Basiri et al., 2014). Hence the sperm cilium is anchored to the nucleus by an extended basal body and develops almost entirely within the cytoplasm in contact with other organelles. This process is termed cytosolic ciliogenesis to distinguish it from the compartmentalised ciliogenesis of primary cilia (Avidor-Reiss and LeRoux, 2015).

The function of transition fibre proteins has been less well studied in sperm flagella compared to primary cilia and the motile cilia of multiciliated cells such as the lung. Since the developing spermatid flagella are accessible to the cytoplasm, there would not appear to be a requirement for a ciliary import mechanism. Furthermore, the structures associated with the ciliary gate in mammalian primary cilia (the transition fibres and transition zone Y-links) have not been identified in *Drosophila* spermatids (Jana et al., 2018). Transition zone proteins are present but these are at the ciliary cap rather than adjacent to the basal body. Additionally, IFT, required for the extension of primary cilia including *Drosophila* type I sensory neurons, is not required for sperm axoneme extension even in the ciliary cap where microtubule polymerisation occurs (Han et al., 2003; Sarpal et al., 2003).

Here we describe the phenotypes of the first *Drosophila* mutants and the effects of RNAi-mediated knockdown for an orthologue of a transition fibre component of the ciliary molecular gate. Twitchy, the *Drosophila* orthologue of mammalian FBF1/*C. elegans* dyf-19, is required for coordination and the production of motile sperm demonstrating that twitchy is essential for the function of both compartmentalised and cytosolic cilia and suggesting a role for twitchy beyond the ciliary gate.

## RESULTS

### Twitchy mutant flies are uncoordinated

**Fig. 1.**
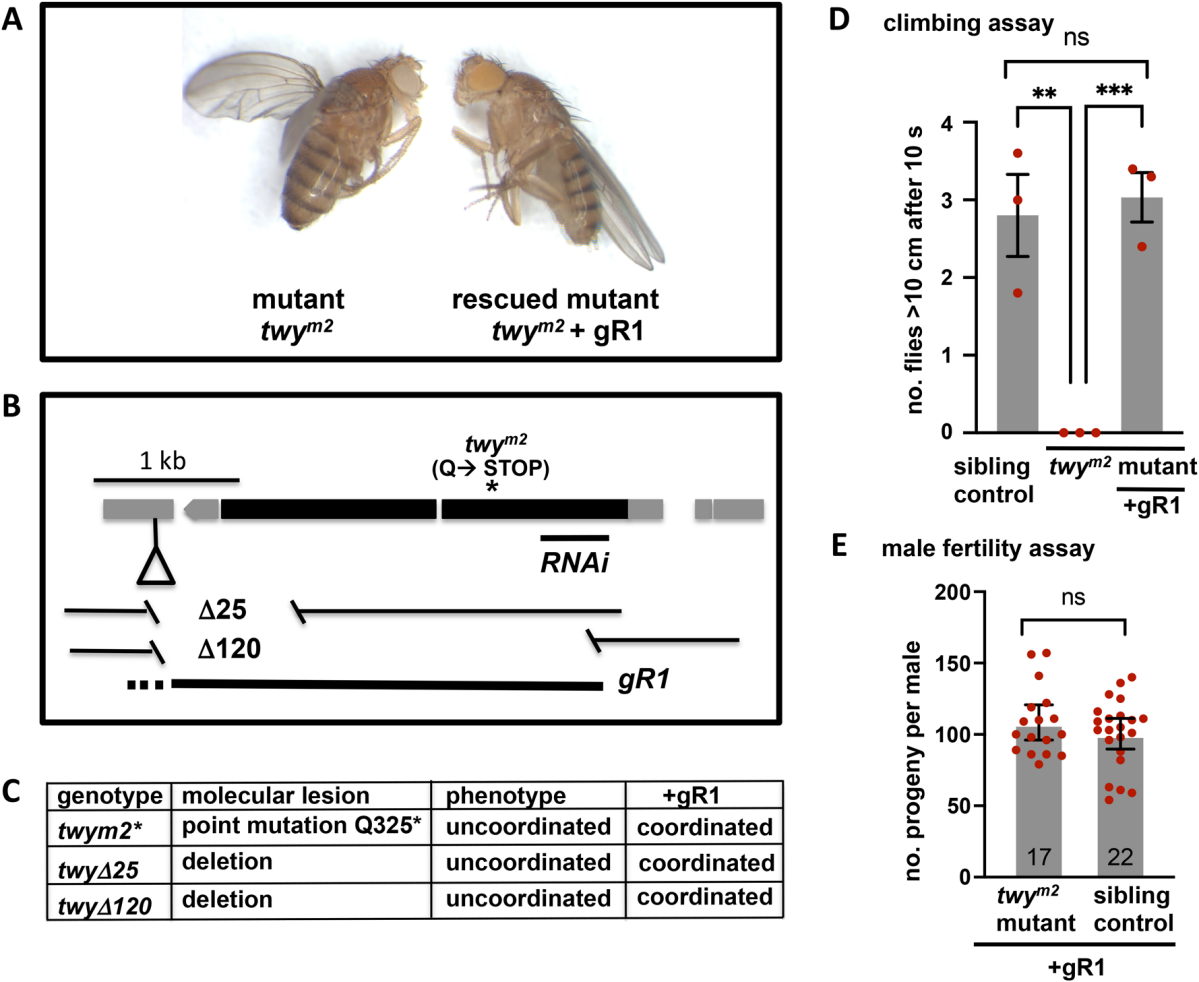
***Twitchy* mutants are uncoordinated.** (A) *Twitchy* adult mutant flies (*twy^m2^*, left) are morphologically normal but uncoordinated. The flies cannot balance and typically show held out wings and twisted legs. Expression of a single wild-type copy of the *twy* gene (*gR1*, right) restores coordination to all *twy* mutant fly lines. (B) Genomic organisation of *twitchy*. Coding regions and untranslated regions are shown as black and grey boxes, respectively. The insertion site of the p-element transposon (P{GawB}Pi3K68D^NP2686^) used to generate deletions is shown as an inverted triangle and the deletions (for mutants Δ25 and Δ120) as gaps. *Twy^m2^* is an ems mutation that results in a C-to-T transition at position 1127 (within the CG5964 gene) that changes Q325 to a stop codon. RNAi denotes the position of the UAS-RNAi knockdown line. gR1 denotes the genomic rescue construct. (C) Each of the molecular lesions generates uncoordinated flies and this phenotype is rescued by the genomic construct, gR1. (D,E) Rescued mutant adult flies show normal climbing ability and fertility. (D) The climbing ability of *twy^m2^* mutants, sibling controls (*twy^m2^*/TM3) and rescued *twy^m2^* mutant+gR1 (gR1/+; *twy^m2^*) males is shown as the number of flies climbing >10 cm in 10 s. Each data point represents the mean of a batch of ten flies tested five times. The mean±s.e.m. is shown for *n*=3 experiments (groups of ten flies). (E) The fertility of rescued *twy^m2^* mutant males (gR1/CyO; *twy^m2^*, *n*=17) is shown as the mean±s.e.m. compared to sibling controls with one mutant copy of the *twy* gene and a wild-type copy on the balancer, TM6B, chromosome (gR1/CyO; *twy^m2^*/TM6B, *n*=22). Males were individually mated with two Canton S females at 25°C. The data points represent the number of progeny per individual male. A *P*-value of 0.3155 from a two-tailed, unpaired *t*-test is not significant (ns).

From a screen for lethal mutations within the cytological region 68D of the *Drosophila* genome, we identified a class of loss-of-function mutants that gave rise to an uncoordinated phenotype. Mutant animals (homozygous or hemizygous over a deficiency) appeared to die during development as the emerging flies stuck to the surface of the food. However, when selected as larvae by their locomotor defect and cultured on agar plates, the newly eclosed adults displayed no apparent morphological defects apart from a partial held-out-wing phenotype (Fig. 1A). Most strikingly the mutant animals were severely uncoordinated. They attempted a characteristic grooming response involving stroking of the wings by their back legs but were unable to balance on their remaining legs during this process. We named the gene ‘*twitchy* (*twy*)’ as mutant adults were able to twitch their legs (Movie 1) but were unable to balance, walk, jump or fly (*n*=>100 animals).

### The locomotor phenotype is not due to a defect at the neuromuscular junction

To determine whether the mutant behaviour was due to a defect at the neuromuscular junction resulting in reduced synaptic transmission and hence muscle activity, evoked and spontaneous transmitter release and quantal content at the neuromuscular junction was measured in third instar larvae (Table 1). Evoked neurotransmitter release and quantal content were not significantly altered in mutant larvae. Although there was an increase in spontaneous events, indicative of an increased probability of release, this was probably not biologically significant as the evoked excitatory junction potentials were not different. This indicated that the twitchy locomotor defect was not due to a defect in motor output.

**Table 1.**
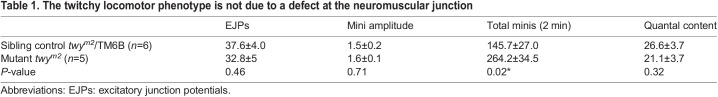
The twitchy locomotor phenotype is not due to a defect at the neuromuscular junction

### Twitchy is the *Drosophila* orthologue of human FBF-1/C elegans dyf-19

Sequence analysis of the *twitchy* mutant alleles (Fig. 1B) identified lesions within the gene CG5964 (Flybase version 2020_05, FBgn0036206, 3L: 11,790,212—11,793,283), the *Drosophila* orthologue of human FBF1 (40% similarity and 22% identity at the protein level) and *C. elegans* dye-filling defective 19 (dyf-19; 36% similarity, 21% identity at the protein level, DRSC integrative orthologue prediction tool). The *twitchy* gene has a single predicted transcript of 3007 nucleotides that encodes a 928 amino acid protein of 106 kDa and contains seven regions predicted to form coils at amino acids: 311-331; 336-356; 473-504; 540-648; 650-677; 690-727; (Interpro features: http://www.ebi.ac.uk/interpro/protein/Q9VTN6) a structure common to many ciliary and centriolar proteins. The ems allele (named twy^m2*^) introduced a premature stop codon within the first exon (Q325→ *; Fig. 1B) and hence would be predicted to prevent any function of the coiled-coil region. Imprecise excision of a P-element generated the deletions *twyΔ^25^* and *twyΔ^120^*. Although neither excision deleted the predicted ATG start codon, the phenotypes were identical, for each allele, for animals homozygous or hemizygous over a deficiency, Df(3L)BSC727, that uncovers the entire *twitchy* gene indicating that these alleles are likely to be genetic nulls/loss-of-function mutants. A single copy of a mini gene (gR1, Fig. 1A,B) rescued the uncoordinated phenotype of each of the *twitchy* mutant alleles (Fig. 1C) and the negative gravitaxis response present in wild type flies (Fig. 1D). The rescued mutant male flies were fertile producing the same number of progeny as sibling controls (Fig. 1E). Hence locomotor activities including those required for mating and climbing could be attributed to loss of twitchy function. Since loss of the *C. elegans* orthologue dyf-19 is associated with defective cilia (Wei et al., 2013), this implicated twitchy in the function of the ciliated sensory neurons that coordinate locomotion. As mutant adult flies were so severely uncoordinated we turned to larvae where movement is less dependent on sensory feedback to further analyse the *twitchy* locomotor defect.

### Sensory behaviour is reduced in *twitchy* mutant larvae

**Fig. 2.**
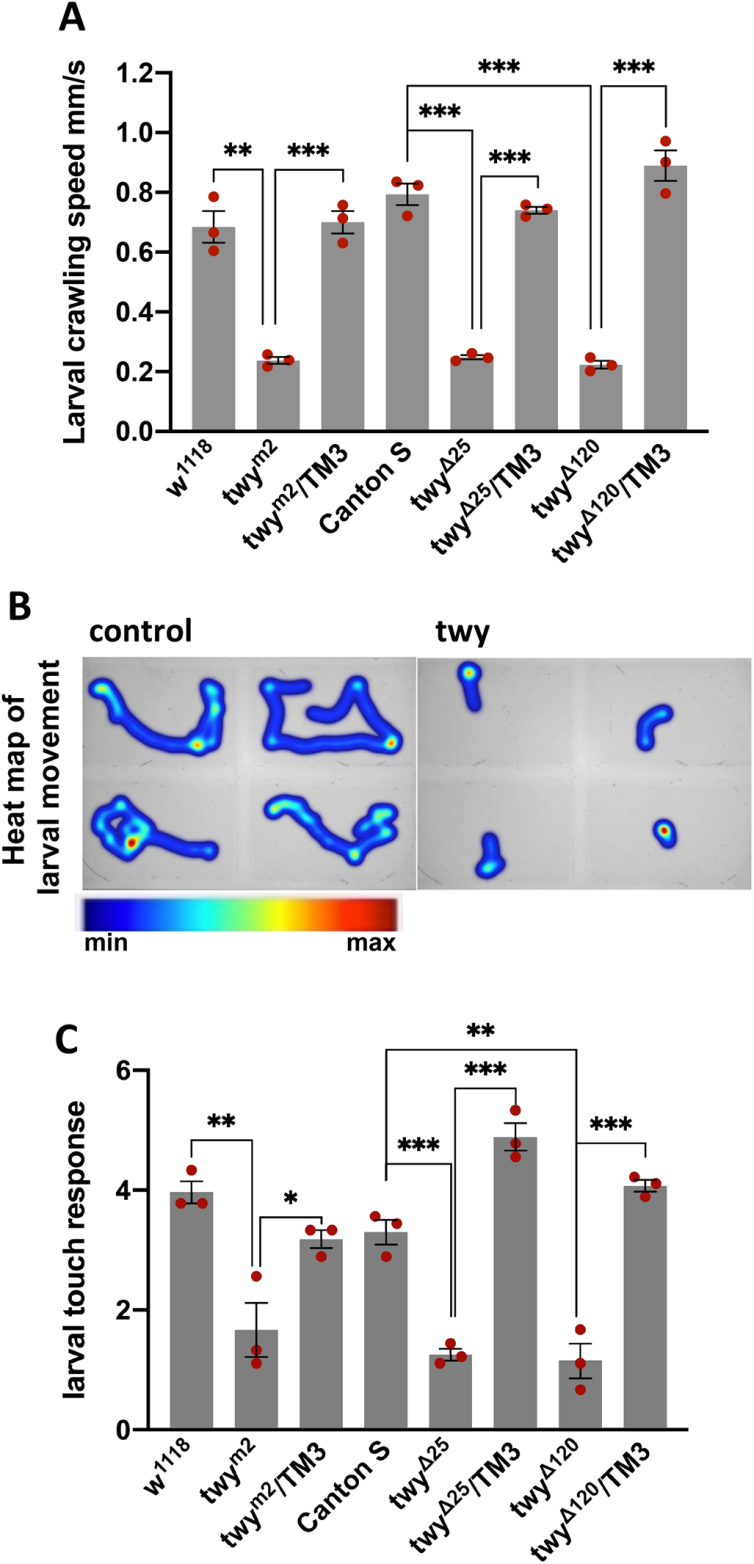
**Sensory behaviour is reduced in *twitchy* mutant larvae.** (A) *Twy* mutant larvae have reduced larval speed. The locomotion of wandering third instar larvae was analysed for the *twy* mutant alleles (*m2*, *Δ25* and *Δ120*), their sibling controls (*m2*/TM3, *Δ25* /TM3 and *Δ120*/TM3) and the parental lines w^1118^ and Canton S. Locomotion was recorded for 3 min for *n*=20 larvae for each genotype per assay and the assay was repeated three times with separate batches of larvae. The mean speed (±s.e.m.) for the three assays is shown. (B) Locomotory behaviour is altered in *twy* mutant larvae. Heat maps are shown of larval movement (recorded in A) representative of mutant and control genotypes. (C) Sensitivity to touch is reduced in *twy* mutant larvae. Larvae (from 2A) were brushed with a human eyelash and their response to touch scored. Each larvae was stimulated three times for *n*=20 larvae and the three scores summed to give a score/larva. The assay was repeated three times. The larval response to touch is shown as the mean score (±s.e.m.) for the three assays.

Throughout larval life mutant animals (homozygous for the *twitchy* mutant alleles or hemizygous over a deficiency for the region) could be distinguished from their siblings (with one copy of *twitchy*) by their locomotor defect. Mutant larvae were slower to crawl and more likely to adopt a ‘C’-shape or curl into a ball. Analysis of wandering third instar larvae on non-nutritive agar plates (Fig. 2A,B) showed that the mean speed of the *twy^m2^* mutant (0.24 mm/s) was significantly reduced compared to wild type (*w^1118^*, 0.685 mm/s, *P*=0.0012) and sibling (*twy^m2^*/TM3, 0.70 mm/s, *P*=0.0003) controls. Similarly, the mean crawling speed of wandering third instar larvae for the imprecise excision alleles *twyΔ^25^* and *twyΔ^120^* was 0.24 mm/s and 0.22 mm/s, respectively, significantly slower than the parental wild-type strain Canton S (0.79 mm/s) and their sibling controls (*twyΔ^25^*/TM3, 0.74 mm/s, *P*=<0.0001; *twyΔ^120^*/TM3, 0.89 mm/s, *P*=0.0002). Crawling behaviour could also be distinguished; whereas control larvae explored the agar plate, turning when about to contact the saline solution at the edge, *twitchy* mutant larvae covered only short distances, frequently pausing and curling into a ball rather than changing direction (Fig. 2B). To look at the response of larvae to an external stimulus, a touch assay was used (Fig. 2C). Mutant larvae (*twy^m2^*, *twyΔ^25^* and *twyΔ^120^*) showed a reduced response to touch compared to the controls lines. Thus coordination of movement in both adult and larvae requires the normal function of twitchy and is likely to involve ciliated type I sensory neurons found in a range of external sensory organs and chordotonal organs.

### Twitchy and sperm production

**Fig. 3.**
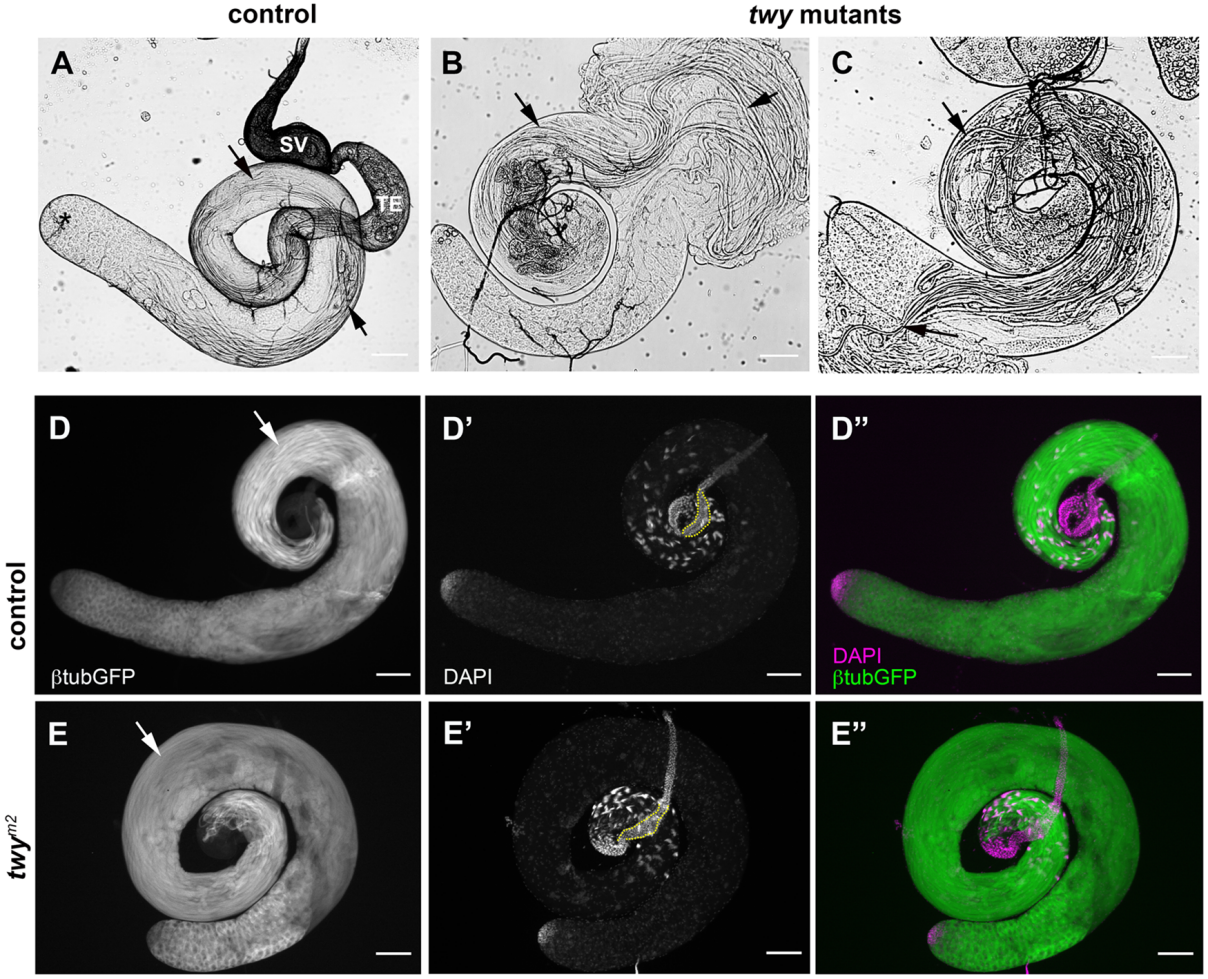
**Mutation of *twitchy* does not affect the gross morphology of the testes.** (A–C): Representative images of testes from control (Canton S, A) and *twy* mutant alleles (*m2* in B and *Δ25* in C) visualised by phase contrast microscopy. (A) Intact control testes showing the different stages of sperm development. The testes are coiled tubes. Spermatogenesis begins at the closed apical end (*) with the division of a germline stem cell to produce a cyst of 64 haploid spermatids. These develop into bundles of spermatids that elongate to fill most of the length of the testes (arrows). Mature sperm individualise and coil up at the basal end of the testes marked by the terminal epithelial cells (TE) then are released and swim from the testes lumen into the seminal vesicle (SV) for storage. In lightly squashed *twy* mutant testes (B,C) from young males, the gross morphology of the testes is unchanged and cysts of elongated sperm bundles can be observed to spill out of the testes on dissection (arrows). Testes are lighter in mutants (B,C) because of differences in genetic background compared to the Canton S control (A). (D,E) Fixed preparations of testes from flies expressing β1-tubulin-GFP marks both axonemal and cytoplasmic β1-tubulin and shows the mature sperm bundles formed in *twy^m2^* mutant flies (β1-tubulin-GFP/+; *twy^m2^*, E,E″) and sibling controls (β1-tubulin-GFP/+; *twy^m2^*/TM6B, D,D″). DAPI staining (D′,E′) marks the nuclei of the mature sperm bundles at the basal end of the testes and the germline stem cells at the apical end. The nuclei of the somatic epithelial cells of the terminal epithelium (see A) and seminal vesicles (outlined by dotted line in D′ and E′) are also visible. The seminal vesicles are empty at this stage (0–1 days). Scale bars: 100 µm.

Since the only cell type apart from type I sensory neurons that contains cilia in flies is the sperm cell, we were interested in determining whether twitchy is required for sperm production and male fertility. The stages of *Drosophila* spermiogenesis leading to mature sperm with their elongated cilia/flagella can be visualised in preparations of testes from freshly dissected adult flies when viewed by phase contrast microscopy (Sitaram et al., 2014). Gentle squashing allows the release of cysts, with their bundles of elongated spermatids, from the testes and motile spermatids from the seminal vesicles of mature males. The testes from *twitchy* mutant flies were of a similar shape and size to controls and their gross morphology appeared normal with the formation of elongated sperm bundles (Fig. 3A–C). These were also visible in fixed preparations of testes expressing β1-tubulin-GFP (Fig. 3D,E) that marks both axonemal and cytoplasmic β1-tubulin (Inoue et al., 2004). Mutant adult flies were short-lived but in young adults although the testes appeared more fragile, elongated sperm extended into the most basal regions of the testes.

### Knockdown of twitchy within ciliated structures affects coordination and fertility

**Fig. 4.**
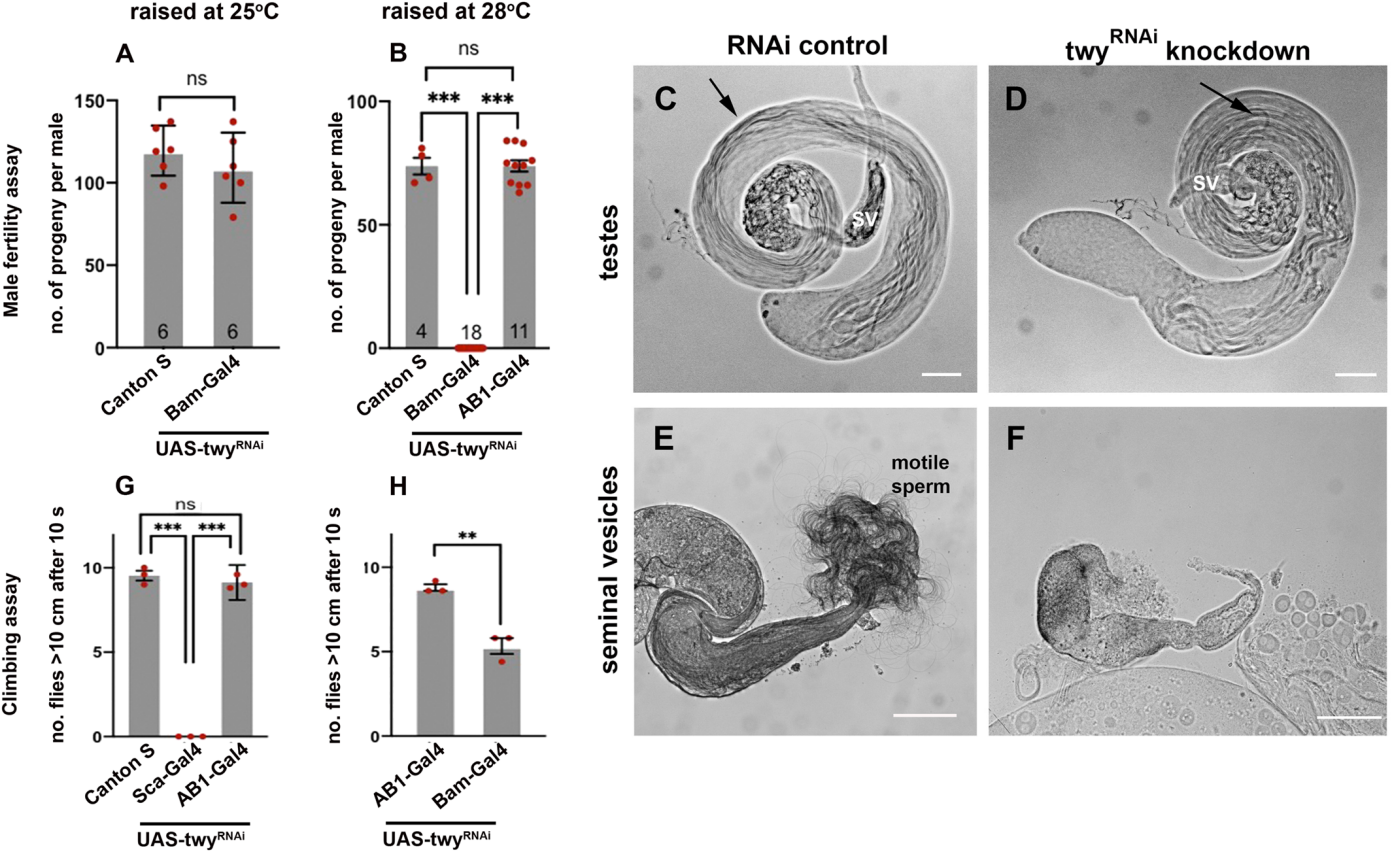
**RNAi-mediated knockdown of twitchy within ciliated structures affects male fertility and coordination.** Knockdown of twitchy RNA with tissue-specific Gal4 drivers was achieved in the testes with Bam-Gal4 (A–F,H) and in the peripheral nervous system with Sca-Gal4 (G). A parental Gal4 line (AB1) and the wild-type Canton S strain were used as controls. Flies were raised at 25°C (A,G) or to enhance expression at 28°C (B–F,H). (A–F) RNAi-mediated knockdown of twitchy within the testes affects male fertility. Males were raised and individually mated with two Canton S females at 25°C (A) or 28°C (B–F). The data points represent the number of progeny per individual male. Male fertility was lower in control lines at 28°C compared to 25°C (compare B with A) but the elevated temperature was required to enhance expression of the twitchy RNAi construct. At 28°C and with two copies of the twitchy RNAi knockdown construct, male fertility was lost (B). Phase contrast images of the male reproductive system of twitchy knockdown and control flies (C–F) showed that mature elongated sperm bundles (arrows) could be seen within the testes (C,D) but the masses of motile sperm released from the seminal vesicle of RNAi controls on gentle squashing (E) was not observed for twitchy-knockdown males (F). (G,H) RNAi-mediated knockdown of the twitchy message within cilia of the peripheral nervous system severely impairs coordination. Knockdown of twitchy mRNA with Sca-Gal4 but not the parental AB1-Gal4 or testes-specific Bam-Gal4 severely impaired coordination and flies were too uncoordinated to effect a climbing response (G). To confirm that the infertility shown by the RNAi-mediated knockdown of twitchy in the testes in flies raised at 28°C (shown in B) was not due to an effect on the nervous system, these flies were also tested for their climbing ability. Flies expressing two copies of twitchy RNAi under the control of Bam Gal4 and raised at 28°C were coordinated and could walk and climb although they showed an impairment of the climbing response compared to the AB1-Gal4 control (H). The climbing ability is shown as the number of flies climbing >10 cm in 10 s. Each data point represents the mean of a batch of ten flies tested five times. The mean±s.e.m. is shown for *n*=3 experiments (groups of ten flies). Preparations in C–F are fixed testes from 0–2 days flies (C,D) and unfixed testes from 8–12 days males kept separate from females (E,F). Scale bars: (C–F) 100 µm.

Since these *twitchy* mutant adult flies were short-lived and severely uncoordinated it was not possible to assess directly whether male fertility was affected. Instead, fertility was assessed following RNAi-mediated knockdown of the twy message (UAS-twy^RNAi^) in the male germline (with Bam-Gal4-VP16) using the Gal4-UAS system (Brand and Perrimon, 1993; White-Cooper, 2012). Male flies of the genotype UAS-dcr2, UAS-twy^RNAi^; Bam-Gal4-VP16/+ were coordinated, consistent with the expression of Bam-Gal4 in the testes. There was no loss of fertility when flies were raised at 25°C (Fig. 4A) but they were infertile when raised at 28°C to enhance Gal4 expression (Fig. 4B). At this temperature, there is a trade-off between enhanced Gal4 expression and a decrease in fertility that is observed (in wild-type flies) at higher temperatures. Hence, the number of progeny produced by control flies at 28°C (74±2 progeny, mean+s.e.m. for *n*=15 males) was lower than that of flies raised and mated at 25°C (120±6 progeny, mean+s.e.m. for *n*=6 males). Although there was some impairment of their climbing ability compared to controls (Fig. 4H), twy-knockdown males were coordinated and observed to mate. The testes from twy-knockdown males produced elongated sperm bundles (Fig. 4D) but the seminal vesicles appeared empty and did not produce the mass of motile sperm seen in gently squashed preparations of control testes on aging (compare Fig. 4E and F).

Knockdown of the twitchy message using the Sca-Gal4 line which expresses within the peripheral nervous system generated uncoordinated flies (*n*=>120 animals) even at 18°C when the temperature-sensitive Gal4 expression is reduced. Consistent with this phenotype, flies with Sca-Gal4 knockdown of the twitchy message did not show a gravitaxis response, assessed by their ability to climb in response to a bang stimulus (Fig. 4G). In contrast, RNAi-mediated knockdown of the twitchy message with Gal4 drivers such as AB1, with no expression in the nervous system, had no effect on climbing ability (Fig. 4G). These results are consistent with twitchy functioning within both the ciliated sensory neurons to allow coordinated locomotion, and in sperm cells to allow the production of motile sperm and male fertility.

### Loss of twitchy results in a defect late in spermiogenesis

**Fig. 5.**
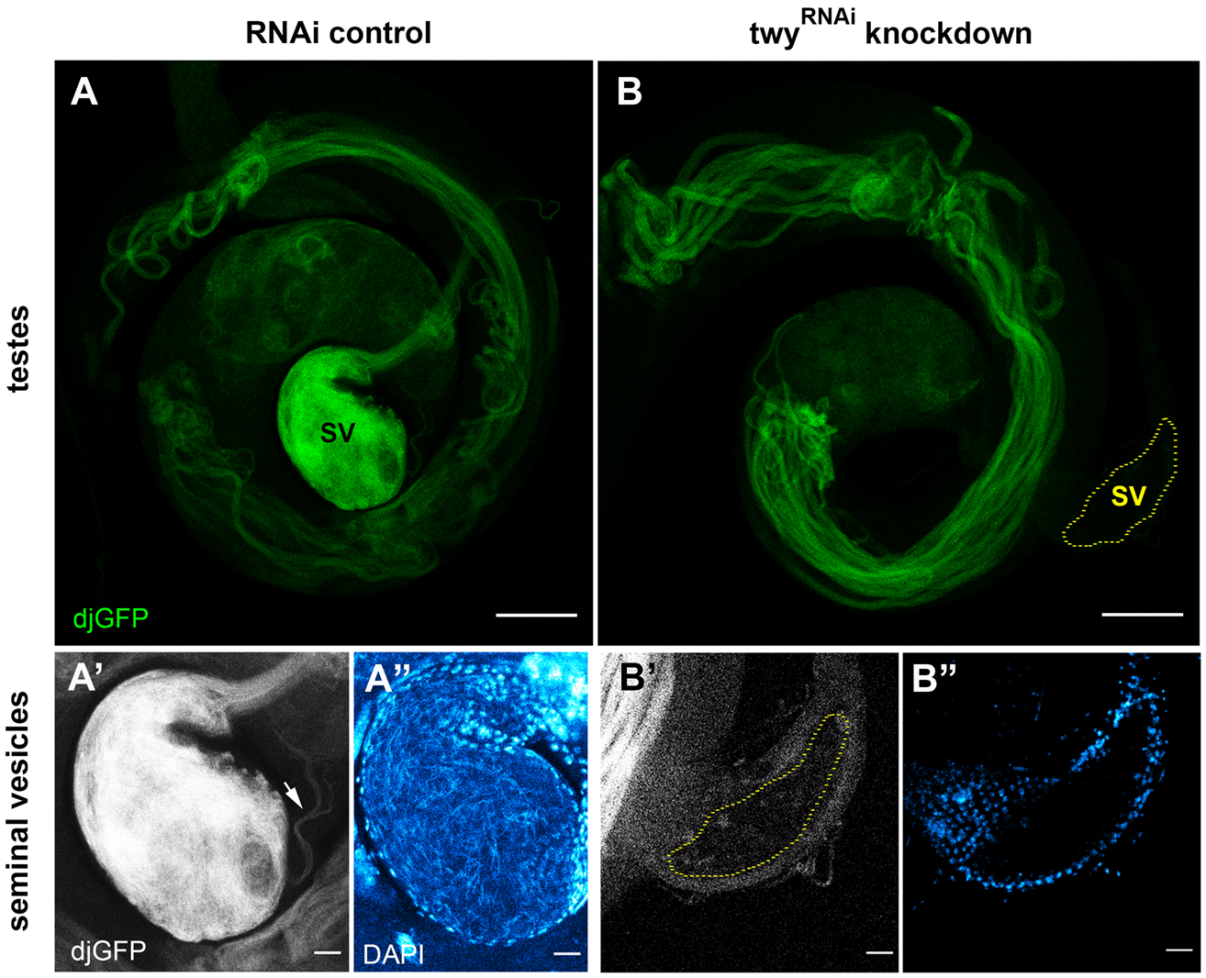
**In twitchy-knockdown flies the sperm elongate but do not migrate to the seminal vesicles.** Representative confocal images of fixed whole mount testes from 10–12 days old control (A) and RNAi-mediated twitchy-knockdown (B) flies. In A, mature elongated sperm, the tails labelled with dj-GFP in green (and in grey in the magnified views of the seminal vesicles in A′) accumulate in the seminal vesicle (SV). In twy^RNAi^ testes (B) elongated sperm are present in the testes but fail to accumulate in the SV. The empty SV is marked by the dashed outline. In the magnified views of the SV, individualised spermatids are clearly visible in control testes as dj-GFP-marked tails in A′ and needle-shaped nuclei marked by DAPI, in cyan, in A″. The spermatids can be visualised moving through the testicular duct (arrow in A′) from the base of the testes into the seminal vesicle. In B″ DAPI labels the round nuclei in the epithelial cells of the outer wall of the SV but the vesicle is empty of spermatids. Dj-GFP in B′ is enhanced to show the background and the empty SV is marked by the dashed line. Scale bars: 100 µm (A,B); 20 µm A′–B″.

To address the sperm defect we looked at sperm from twitchy-knockdown males as, unlike their mutant counterparts, these flies were coordinated and hence viable as adults and could be aged. The sperm made in the testes accumulate in the seminal vesicles for release during mating hence the seminal vesicles become enlarged over time in unmated males. The testes-specific marker don juan (dj)-GFP (Santel et al., 1997) labels mitochondria in elongated spermatids in the testes and seminal vesicles (Fig. 5A). When twitchy expression was reduced through RNAi-mediated knockdown, dj-GFP staining was retained in the elongated spermatids in the testes (Fig. 5B) consistent with the presence of cysts of elongated spermatids visualised by phase-contrast microscopy (in Fig. 4D). However, these knockdown sperm failed to accumulate in the seminal vesicles on aging (Fig. 5B) consistent with an impairment in the production of motile sperm. As mitochondria but not the tubulin axoneme is essential for sperm tail formation (Noguchi et al., 2011) we confirmed the presence of the axoneme using antibodies to modified tubulin.

**Fig. 6.**
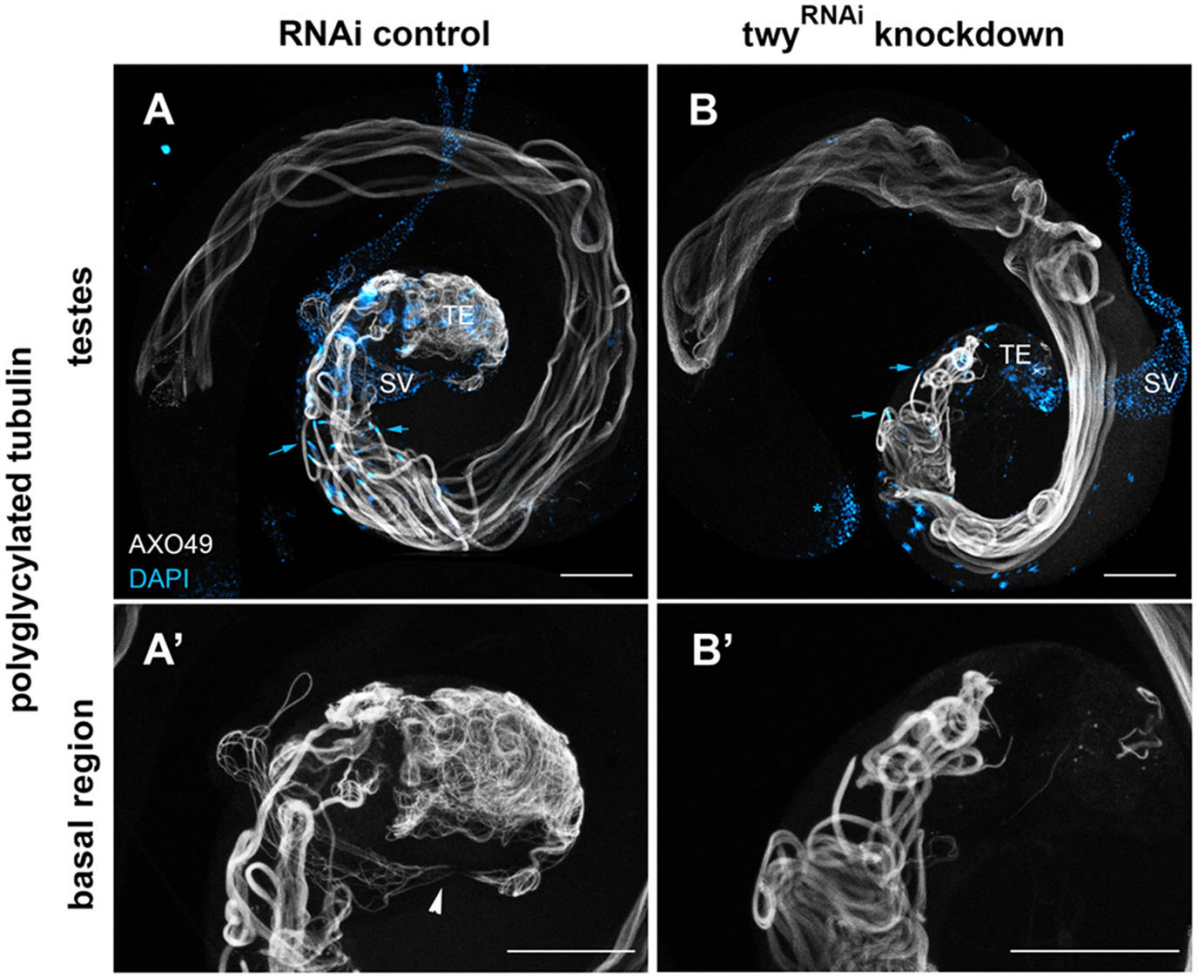
**Twitchy is not required for modification of the tubulin axoneme by polyglycylation.** Representative confocal images of whole mount testes from 2-day-old control (A,A′) and RNAi-mediated twitchy-knockdown (B,B′) flies stained with AXO49, show polyglycylation (in grey) of axonemal tubulin in fully elongated spermatids of both control and twitchy-knockdown testes. The twitchy-knockdown testes lack the mass of individualised spermatids seen within the most basal portions of the control testes (terminal epithelium region, TE). In the magnified views (A′,B′), individualised spermatids can be visualised moving through the testicular duct (arrowhead in A′) from the base of the testes into the seminal vesicle in the control testes. DAPI staining (shown in cyan) marks the nuclei of the spermatids (arrows indicate nuclei of spermatid bundles in A,B) as well as the epithelia of somatic cells such as the seminal vesicle (SV, in A,B) and germline stem cells at the apical region (visible in the testes in B, *). The diffuse staining in the terminal epithelium region in the DAPI channel is autofluorescence. Scale bars: 100 µm.

During spermiogenesis, tubulins are post-translationally modified, a process important for axoneme stability and motility (Rogowski et al., 2009; Wloga et al., 2017). Following polyglutamylation and detyrosination, the fully elongated axonemes are polyglycylated at the onset of individualization and mutants in the two *Drosophila* glycylases and a putative glycine transporter (Chatterjee et al., 2011) are sterile and lack motile sperm. Polyglycylation of tubulin can be seen in the fully elongated axonemes of spermatid bundles and individualised spermatids in the testes of control flies (Fig. 6A). In twitchy-knockdown testes, the mature elongated spermatid bundles were polyglycylated (Fig. 6B) indicating that twitchy is not required for this process. However, whereas masses of fine individualised spermatids were observed at the base of the testes in control testes (Fig. 6A′) these were largely absent from twitchy-knockdown testes (Fig. 6B′). These results from younger 2-day-old flies together with those from aged males (Figs 4F and 5B) are consistent with a lack of motile sperm formation in twitchy-knockdown males. This might arise from a defect in the generation of individualised sperm.

**Fig. 7.**
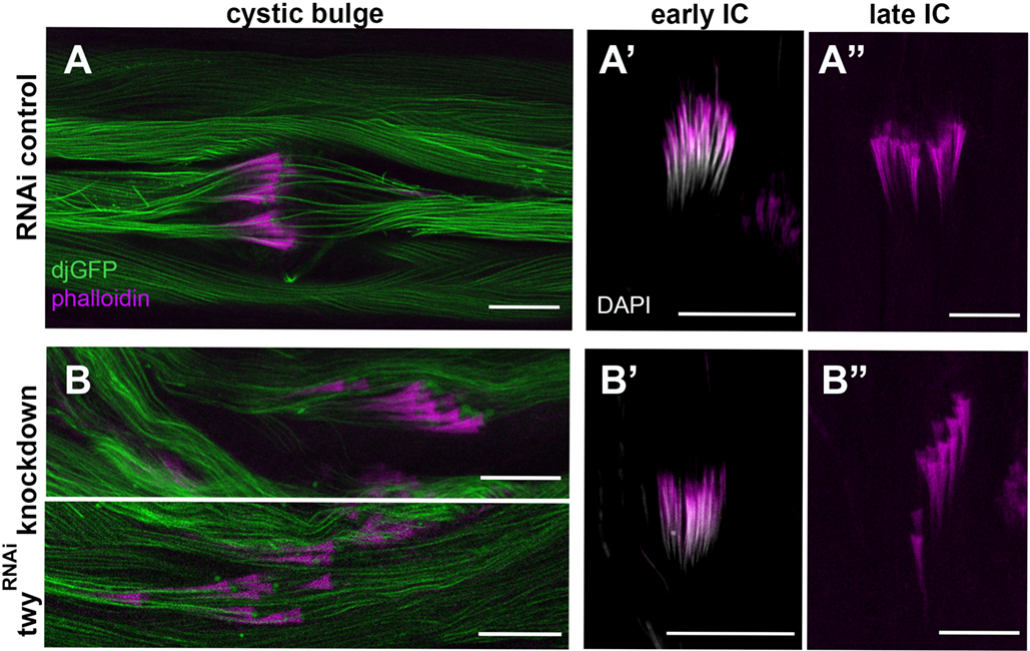
**Spermatid individualisation in twitchy-knockdown flies.** Representative confocal images of testes from 0–2 days old control (A) and RNAi-mediated twitchy-knockdown (B) flies. During individualisation, actin-rich complexes marked by phalloidin (magenta) form around the nuclei (labelled with DAPI, grey) initially as needle-shaped structures (early ICs, A′) then as cone-shaped structures (A″) that progress away from the nuclei along the spermatid tails generating a cystic bulge (A) containing waste cytoplasmic contents. In RNAi-mediated twitchy-knockdown flies, the actin individualisation cones form around the nuclei (B′) but mainly fail to remain synchronised as they progress (B,B″). In B, two sections of the same testes show separate clusters of actin cones. Spermatid tails are marked by dj-GFP. Scale bars: 20 µm.

Individualisation of mature spermatid bundles occurs following tubulin modification. Actin-rich complexes form around each of the 64 nuclei within a cyst and migrate synchronously along the length of the elongated spermatids removing unneeded cytoplasmic contents into a cystic bulge (Fig. 7A) that is discarded in a waste bag at the spermatid tail. To determine if individualization might be disrupted by loss of twitchy, we looked at the formation of the actin-rich individualization cones by staining with phalloidin (Fig. 7). Individualization complexes formed normally around needle-shaped nuclei in both control (Fig. 7A′) and RNAi-mediated twitchy knockdown (Fig. 7B′) testes and examples of their progression to cone-shaped structures could be found. However, in twitchy-knockdown flies, there were fewer intact complexes and waste bags at the apical ends of the testes, actin cones were frequently scattered (Fig. 7B″) and the flagella around the cystic bulge tended to be more disorganised (Fig. 7B). The structure of the actin cones was also often abnormal appearing narrower or occasionally shorter presumably from the loss of their front edges or rear actin bundles, respectively. Although these effects were also observed for some control testes, possibly because of the negative effect of increased temperature on the individualization process (Ben-David et al., 2015) and fertility, they were more pronounced in twitchy-knockdown flies. Therefore, in twitchy knockdown flies the spermatid flagella, including the microtubule axonemes, assemble, elongate and are post-translationally modified. Some aspect of the individualisation process, or a prior step required for individualization to occur, may, however, be defective resulting in a lack of motile sperm that fail to migrate to the seminal vesicles.

## DISCUSSION

### Twitchy is an evolutionary conserved protein required for specialised ciliary function

We have identified mutations in the *Drosophila* orthologue of human *FBF1/C. elegans dyf-19*, the first mutations reported in flies for a molecular component of the transition fibres. The uncoordinated phenotype and loss of sperm motility are consistent with a function for twitchy in each of the ciliated cells found in flies namely the type I ciliated sensory neurons of the external sense organs and chordotonal organs, and sperm cells. The uncoordinated *twitchy* phenotype is consistent with the sensory phenotype of *dyf-19* mutants (Wei et al., 2013). In adult flies locomotion is coordinated principally by external mechanosensory organs especially the bristle cells while the chordotonal organs act as internal proprioceptors and sense gravity and sound. The severity of the *twitchy* uncoordinated mutant phenotype suggests that mechanotransduction in bristles (external sense organs) is defective (Enjolras et al., 2012; Kernan et al., 1994) since adult flies lacking chordotonal organ function only are sedentary but sufficiently coordinated to walk, climb and mate (Eberl et al., 2000; Kavlie et al., 2010; Ma and Jarman, 2011). The reduced touch sensitivity of *twitchy* mutant larvae, however, indicates that chordotonal organ function is also defective in *twitchy* mutants. A role for twitchy in all ciliated sensory cells would be consistent with a function in gating the movement of large molecules into and out of the cilia required for its assembly and function. The RNAi-mediated knockdown of the twitchy message in the male germline impaired the production of motile sperm and resulted in infertile males. Hence twitchy was not required for the formation or elongation of the sperm ciliary axoneme but for some aspect of its maturation or function. The requirement for twitchy in sperm cilia indicates that the function of twitchy /FBF1 may extend beyond the ciliary gating role established for FBF1/dyf-19 in primary cilia (Tanos et al., 2013; Wei et al., 2013; Yang et al., 2018).

### Twitchy is required for compartmentalised and cytosolic ciliogenesis

Our results show that twitchy is required for the function of cilia formed by both compartmentalised ciliogenesis (sensory functions) and by cytosolic ciliogenesis (motile spermatids). By analogy with the function of FBF1 and dyf-19 we would anticipate that twitchy functions in the active import of cargo including IFT complexes for the assembly and function of the *Drosophila* type I sensory neuron cilia. However, IFT is not required for the extension of the microtubule axoneme in *Drosophila* sperm cells even in the ciliary cap where the polymerisation of tubulin takes place (Han et al., 2003; Sarpal et al., 2003). It has recently been shown that certain axonemal proteins, such as dyneins, can be locally translated and incorporated into the axoneme directly from the cytoplasm (Fingerhut and Yamashita, 2020). Hence, there seems to be no requirement for an import machinery to move cargo across a compartment boundary. This suggests that in sperm cilia, twitchy plays a different role to its presumed function in ciliary gating in compartmentalised cilia.

In mammalian cells, five molecular components of the transition fibres have been identified from proteomic studies (Tanos et al., 2013) but they have mainly been studied in primary cilia and the smaller motile cilia of multiciliated cells. Hence their role in sperm flagella and cytosolic ciliogenesis is unclear. In the mouse, Cep164 is highly expressed in spermatids and a conditional knockdown results in male infertility (Devlin et al., 2020; Siller et al., 2017) indicating that transition fibre proteins may also act together in sperm cilia as they do in other types of cilia. Only FBF1 (twitchy), Cep89 and Cep164 have clear orthologues in flies (on the basis of reciprocal BLAST searches, Wei et al., 2015). Loss of *Drosophila* Cep89 mRNA by RNA-interference generates a locomotor defect (van Bon et al., 2013) consistent with a role in ciliated sensory neurons but the effects of Cep89 knockdown on sperm function were not investigated. However, high throughput expression data (modENOCODE RNA-Seq, Flybase) indicates that both Cep89 and twitchy mRNA are present in the testes, consistent with a role for multiple transition fibre proteins in sperm.

It is clearly now important to establish the nature of the sperm defect in *twitchy* mutants, for example whether it is required for some aspect of individualisation, coiling or in maturation and acquisition of motile characteristics, the localisation of twitchy within developing sperm and to identify its molecular interactions. These properties will help to establish how the molecular components of ciliary transition fibres function in the flagella of male gametes and in the process of cytosolic ciliogenesis.

## MATERIALS AND METHODS

### *Drosophila* fly stocks

Stocks were maintained according to standard procedures. The following stocks were obtained from the Bloomington Stock Centre (BL), the Vienna *Drosophila* Research Centre (v), the *Drosophila* Genome Research Centre, Japan (DGRC), from colleagues at Manchester or generated in this study as described below. Deficiencies that uncover the *twy* locus Df(3L)vin3 (68C5-6–68E3-4, BL2609), Df(3L)ED4470 (68A6–68E1, BL8068), Df(3 L)BSC676 (68D6–69A2, BL26528) and Df(3L)BSC727 (68D3–68F2, BL26579). To generate *twy* excisions, the P-element NP2686 (DGRC 104288) was used. For RNAi-mediated knockdown of *twy*, the UAS-line KK104108 (v107853) was used with the Gal4 lines Sca-Gal4, BamGal4-VP16 and AB1 Gal4 (BL1824) and with UAS-dcr2 (BL24644) to enhance knockdown. β1-tubulin-GFP (DGRC 109603) labels both axonemal and non-axonemal tubulin and was used to visualise cellular structures within the testes. Dj-GFP (from BL58406) was used to visualise mature spermatid tails. The lines Canton S (parental line for the P-element NP2686 and thus *twy* excision alleles) and *w^1118^* (the parental line for the *twy* allele, *twy*^m2^) were used as wild-type controls.

### Generation of *twy* mutant alleles

The *twy*^m2^ allele was generated through random chemical mutagenesis with ethyl methane sulfonate. Alleles were selected on the basis of their (adult) lethal phenotype with Df(3L)vin3 (68C5-6–68E3-4) that could be rescued with a minigene (gR1). This construct contained all of the CG11621 coding sequence and 3.1 kb of CG5964. Sequence analysis of *twy*^m2^ identified the introduction of a premature stop codon (Q_325_→* nonsense mutation) within the gene CG5964 at position 1127. Further loss of function alleles, *twy*^NPΔ25^ and *twy*^NPΔ120^ were generated through imprecise excision of a P-element inserted at the genomic locus 68D6 (P{GawB}Pi3K68D^NP2686^). Alleles were selected on the basis of their lethality with twy^m2*^ and rescue with the minigene, gR1. Sequence analysis of *twy*^NPΔ25^ and *twy*^NPΔ120^ identified deletions within the CG5964 gene. Mutant alleles gave the same adult lethal, uncoordinated phenotype when homozygous or hemizygous over deficiencies uncovering the 68D locus: Df(3L)ED4470, Df(3L)BSC676 and Df(3L)BSC727.

All *twy* mutant stocks were maintained using a TM3, Gal4-twi, UAS-EGFP, Sb, Ser balancer, or a TM6B, Tb, Hu balancer. This also allowed the selection of homozygous mutant larvae by the absence of GFP or by the non-tubby phenotype. To obtain adult flies, mutant larvae were cultured in vials and pupae transferred to apple juice-agar plates to eclose.

### RNAi-mediated knockdown of twitchy

Knockdown of CG5964 mRNA was achieved with the Gal4-UAS system (Brand and Perrimon, 1993) for targeted expression of a UAS-*long hairpin* RNAi line, KK104108 (referred to as twy^RNAi^) obtained from the Vienna *Drosophila* Research Centre (Dietzl et al., 2007). UAS-dcr2 was included to enhance knockdown. Crosses were performed at 25°C or for reduced expression where uncoordinated flies were generated with Sca-Gal4, at 18°C. For knockdown within the testes with Bam-Gal4, crosses were performed at 28°C for maximum expression of the RNAi construct and two copies of the RNAi line were used.

### Electrophysiology

Standard sharp electrode recordings (30 MΩ, 3 M KCl) were made from muscle 6 in abdominal segments A2 to A4. Signals were amplified using an AxoClamp, 1322A digitiser and pClamp10 (all from Molecular Devices, CA, USA). The external saline used was HL3.1 (Stewart et al., 1994).

### Behavioural assays

#### Larval crawling

Larval locomotion was recorded at room temperature using an automated tracking system (Noldus EthoVision XT, v11.0) on 2% non-nutrient agar arenas (125×79 mm) separated by 5 M NaCl channels. Individual third instar larvae were washed in distilled water, transferred to an agar arena, allowed to acclimate then recorded for 3 min. Larvae that fell off the arena or paused to investigate the NaCl solution for more than 25 s were excluded from the analysis. For each genotype, 20 larvae were recorded per session and the assay was repeated a further two times with flies generated in independent crosses for each assay (*n*=3 experiments; three groups of 20 larvae; 60 larvae per genotype).

#### Larval touch assay

Individual third instar larvae from the larval locomotion assay were tested for their sensitivity to touch during bouts of linear locomotion at room temperature on 2% non-nutrient agar plates (modified from Caldwell et al., 2003; Chen et al., 2015). Each larva was brushed gently with a human hair moving away from the anterior, starting at the midsection. The touch stimulus was repeated three times per larva with at least 30 s between each stimulus. For each stimulus, larvae with no reaction were assigned 0, larvae that stopped but didn't move away from the hair were assigned 1, larvae that moved away at an angle of <90° were assigned 2 and larvae that moved away at an angle of >90° were assigned 3. The scores for each of the three stimuli were combined to produce a total score between 0–9. For each genotype, ten larvae were stimulated per session and the assay was repeated a further two times (*n*=3 experiments; three groups of ten larvae; 30 larvae per genotype).

### Adult climbing assays

For the climbing assay (modified from Chen et al., 2015), batches of ten flies/genotype were placed in a sealed, 20.5 cm tube allowed to acclimate and, after tapping to the bottom of the tube, their behaviour was recorded and the number of flies passing 10 cm in 10 s was determined. For each batch of flies, the assay was repeated five times and three separate batches of flies were tested (*n*=3 experiments; three groups of ten flies; 30 flies per genotype). For the RNAi-mediated knockdown of twitchy, crosses producing severely uncoordinated progeny were performed at lower temperatures (18–20°C) to reduce expression and pupae were selected and allowed to eclose on apple juice-agar plates as for *twy* mutants. All climbing assays were performed at 25°C.

### Male fertility assays

To determine whether twitchy is required for male fertility, UAS-twy^RNAi^ was expressed in the male germline with Bam-Gal4-VP16 (White-Cooper, 2012) at 28°C. Adult males (0–2 days old) of the genotype UAS-dcr2; UAS-twy^RNAi^; Bam-Gal4-VP16/+, were individually crossed to two wild-type (Canton S) females and allowed to deposit eggs for 5 days. Adult (parent) flies were then removed and all resulting progeny counted. Vials in which any of the parent flies had died before their removal or where vials went mouldy before removal of parent flies were excluded from the analysis of the number of progeny. Males from the wild-type Canton S strain and from crosses with UAS-twy^RNAi^ and the parental Gal4 line AB1, where expression is limited to the salivary glands, were used as controls.

Fertility assays for rescue of *twy^m2^* mutants were performed at 25°C by crossing individual males of the genotype (gR1/CyO; *twy^m2^* or gR1/CyO; *twy^m2^*/TM6B sibling controls) to two Canton S females.

### Testes preparation and imaging

Preparations from 10–20 testes/genotype were analysed. To analyse morphology, testes were dissected from young (0–2 days) adult flies. Where the release of mature sperm from the seminal vesicles was assessed, male flies were separated from females for >2–3 days to allow complete maturation of the sperm before dissection*.*

### Live imaging of testes

To examine gross morphology (Sitaram et al., 2014; White-Cooper, 2012), testes were dissected in PBS and squashed lightly using a coverslip. The preparations were examined using differential interference contrast (DIC) or phase contrast microscopy on a Leica DM6000B microscope and processed using Leica MM AF Premier software.

### Imaging of fixed testes

Testes were dissected in 1X PBS, fixed for 30 min to 1 h in 4% paraformaldehyde (Agar Scientific, UK)/1X PBS, washed in PBS/0.1% TX-100 for at least 1 h and incubated overnight in Vectashield with DAPI (Vector Labs) before mounting.

For phalloidin staining to visualise actin cones, testes were dissected, fixed and washed as above then incubated with TRITC-phalloidin for 45 min at room temperature. Samples were extensively washed in 1X PBS/0.3% TX-100 before addition of the mountant.

### Immunostaining of testes

For whole mount immunostaining, testes were fixed and washed as above. The samples were then blocked in BBX (PBS/0.3% TX-100/1% BSA) for 30 min to 1 h prior to incubation in mouse AXO49 anti-polyglycylated tubulin (MABS276, Merck) diluted 1/200 in BBX for 2 h at room temperature or overnight at 4°C. Following three washes for 15 min each in 1X PBS/0.3% TX-100, testes were incubated in the secondary antibody (FITC-conjugated goat anti-mouse IgG, Jackson ImmunoResearch, Stratech) diluted in 1X PBS/0.3% TX-100 for 2 h at room temperature. The samples were then washed for three times for 15 min each in 1X PBS/0.3% TX-100 before addition of the mountant.

Images were acquired using a Leica TCS SP8 laser scanning confocal microscope (or for β1-tubulin GFP a Leica DM6000B microscope) and analysed using Fiji-ImageJ. Figures were assembled in Adobe Photoshop CS6.

### Statistical analyses

Statistics were performed in Graph Pad Prism 8. Error bars represent standard error of the mean and a two-tailed unpaired Student's *t*-test was used to determine the statistical significance: ns, not significant; *P<0.05; **P<0.01; ***P<0.001.
